# May DNA analyses be biased by hidden oxidative damage? Voltammetric study of temperature and oxidation stress effect

**DOI:** 10.1371/journal.pone.0305590

**Published:** 2024-06-14

**Authors:** Oskar Szczepaniak, Marta Ligaj

**Affiliations:** 1 Department of Biochemistry and Biotechnology, Poznań University of Life Sciences, Poznań, Poland; 2 Department of Industrial Products and Packaging Quality, Poznań University of Economics and Business, Poznań, Poland; Hamadan University of Medical Sciences, ISLAMIC REPUBLIC OF IRAN

## Abstract

The analysis of nucleic acids is one of the fundamental parts of modern molecular biology and molecular diagnostics. The information collected predominantly depends on the condition of the genetic material. All potential damage induced by oxidative stress may affect the final results of the analysis of genetic material obtained using commonly used techniques such as polymerase chain reaction or sequencing. The aim of this work was to evaluate the effects of high temperature and pH on DNA structure in the context of the occurrence of oxidative damage, using square-wave voltammetry and two independent research protocols. We resulted in visible oxidation damage registered in acidic conditions after the thermal denaturation process (pH 4.7) with changes in the intensity of guanine and adenine signals. However, using phosphate buffer (pH 7.0) for DNA denaturation negatively affected the DNA structure, but without any oxidized derivatives present. This leads to the conclusion that oxidation occurring in the DNA melting process results in the formation of various derivatives of nucleobases, both electrochemically active and inactive. These derivatives may distort the results of molecular tests due to the possibility of forming complementary bonds with various nucleobases. For example, 8-oxoguanine can form pairs with both cytosine and adenine.

## Introduction

Nucleic acids are primary research subjects in genetics, molecular biology, and related life sciences. DNA-based detection methods are applied in numerous cases, from medical trials to food security investigations. We can surely say that we live in a DNA era. However, various environmental and physical factors make nucleotides liable for decay [1, 2]. Thus, special methods and approaches are needed to limit the damage to nucleotides after their extraction. The methods applied differ depending on the chemicals used in the preliminary analysis stage and the final purpose of the study [3–5].

Such a variety of factors could negatively affect DNA structure right after its lysis of cells due to exaggerated oxidative stress in the lysis buffer [6, 7]. The point mutations generated at this stage are constant and could extend, resulting in a wide variance in quantitative results and a misinterpretation of data in qualitative analysis [6, 8]. Further, DNA degradation occurs, especially in the melting process, used in numerous repetitive cycles in numerous analytical procedures, i.e., in polymerase chain reaction (PCR), Southern blot, or sequencing [8]. In the mentioned assays, the obtained results are especially affected by damage to the tested isolates.

The most popular method of preliminary control of isolated DNA is a UV-spectrometry measurement at analytical wavelengths of 230, 260, and 280 nm [9, 10]. This fast-check tool is routinely done between the isolation and amplification processes. Theoretically, each oxidation or other damage to DNA structure affects the spectra at 260 nm [9, 11]. In fact, point mutations could hardly be registered using this method, since it aims only to validate the presence of proteins or other impurities in the lysates [12]. Therefore, damage can be observed right after the electrophoresis stage, which is a final step for most protocols of DNA analysis. This hardly helps to identify or analyze of the root cause of the damage in the entire analytical procedure.

Another possible approach is to analyze DNA damage in the tested cells without the lysis process. To mark breaks and other damages to DNA special staining indicators are used, e.g. H2A.X antibody, neutral red, or propidium iodide [13, 14]. The method of measurement is based on the addition of the indicator or staining the tested cells with the compound that interacts with (un)damaged DNA resulting in a characteristic wavelength emission, which can be registered using a microplate reader or fluorescence microscope [15, 16]. This kind of methods can be used as a quick determination for all kinds of genotoxicity assays. However, the evaluation of a group of compounds or compounds with wavelength absorption similar to that of the indicator may bias the results of the study and the dose-response relationship. Moreover, the staining tests often result in highly deviated results, which decrease the scientific soundness of the observed effects [17–19].

One of the eldest and most trusted methods is HPLC-MS/MS determination of the tested nucleotides along with detection of 8-oxoguanine (8-oxo-7,8-dihydro-2′-deoxyguanosine, 8-oxoG), treated as an indicator of DNA oxidative damage [20]. This method is commonly applied in the detection of DNA oxidation in numerous cell line-based studies and provides information about the quantitative damage ratio to DNA. It also confirms the presence of other oxidized derivatives of nitrogen bases, such as hypoxanthine or the presence of adducts formed in radical reactions. However, in the official assay performed by the European Standards Committee on Oxidative DNA damage, this method was considered as low reproducible due to the wide range of results from different laboratories and variants of the method used [21].

Thus, a need arises to apply a fast screening tool, which may be applied right after isolation and amplification. Such a helpful and quick tool may be electrochemical sensors or biosensors [22]. The first report on electroactivity of DNA bases was published in 1960 [23]. Currently, it is known that electrochemical oxidation signals of guanine (G), adenine (A), cytosine (C), and thymine (T) can be observed on voltammograms [24, 25]. The location on the potential axis of these signals depends on the measurement conditions, mainly pH [25, 26]. In typical DNA voltammograms, usually only purine signals are visible [27, 28]. The most oxidizable base is guanine and its signal appears as the first one. Voltammetric measurements allow monitoring damage to nucleic acids caused by physical and chemical factors based on changes in DNA signals [29]. Commonly considered are the guanine, adenine, and 8-oxoG signals [20, 26, 30]. Electrochemical sensors have a fundamental advantage over the other methods used for the determination of 8-oxoG as they allow the examination of DNA samples without hydrolysis, a process necessary when using other methods that can cause additional damage, leading to false results [20, 28, 31].

The DNA sensors may be divided into four categories: label-free, reagent-less; label-free, reagent-dependent; labeled, reagent-less, and labeled, reagent-dependent [32]. The first two categories base on electrochemical detection of the analyte hybridized to the outer layer of the electrode. The reagent-dependent probes need the presence of the electroactive intermediate that binds to the hybridized DNA and generates a specific electrochemical signal registered by the measuring electrode and compared to the background signal measured in the absence of the intermediate. As the mechanism is similar to ELISA tests, the results given by the sensor may be biased positively by side interaction of the intermediate with an unknown compound or impurity, analogously to in the enzyme method.

The first two mentioned types of DNA sensors have already been applied as a part of ready analytical instruments available on the market. [32]. However, costs of the instrument, accessories, and specially designed consumables may be not affordable for a small or state-funded lab. The application of self-prepared budget solutions seems optimal also for researchers from high-income countries. The working electrodes used as the basis of the DNA sensors are usually made of either gold or carbon materials. The latter involves a glassy carbon electrode, pencil graphite, or walled carbon nanotubes (WCNTs) [33]. Depending on the working-electrode used, DNA sensor has a very low detection limit, ranging even less than 0.5 fM for the carbon electrode covered with the complex of gold and nitrogen [34]. However, HPLC-ECD detection based on the electrochemical signal of 8-oxoguanine warranted lower deviation of the results [35]. The other method is the GC-MS/MS detection of hydrolyzed nitrogen bases or their mutated analogs. This method has two potential sources of negative bias, i.e. hydrolysis of the bases and their derivatization stages, as both cannot occur in full yield [36].

Depending on the variant of the voltammetry method used, composition of the working electrode, pH of the tested sample, and whether the measurement is direct or with the help of an intermediate compound, signals of both nucleobases, and their derivatives may be recorded in different potential regions, which complicates the process of their identification in the performed analysis. The presented manuscript proposes an analytical approach that enables the electrochemical detection of dsDNA structure damage that occurs during thermal denaturation, commonly used in molecular biology. The aim of these studies was to determine the oxidative damage resulting from the oxidation of guanine to 8-oxoguanine. Therefore, in preliminary tests, square wave voltammetry (SWV) measurements were made for two synthetic oligonucleotides, one of which was modified by 8-oxoG instead of one guanine. Comparison of the potential and current of the registered SWV signals allowed to determine the location of the 8-oxoG, guanine and adenine signals in both fragments, which facilitated the analysis of damage in DNA samples, occurring after denaturation and exposure to oxidative stress. The results of this study could help prevent errors in molecular studies and develop DNA sample preparation protocols.

## Materials and methods

### Tested material

The tested were two oligonucleotides (Future Synthesis, Poznań) with a sequence:

- 5’ GTCAACTTCCGTACCGAGC (Bar3),
- 5’ GTCAACTTCC-8oxoG-TACCGAGC (Bar3-8oxoG)

as well as calf thymus dsDNA (ctDNA) (Sigma-Aldrich, Poland). The tested samples were dissolved in two different buffers:

- 0.2 M acetate buffer with 0.01M KCl, pH 4.7
- 0.05 M phosphate buffer with 0.01M KCl, pH 7.0.

The samples were then directed to physical treatment, which was either melted at 100°C or H_2_O_2_ at a final concentration of 1.5% in the sample. The chemicals needed for buffer preparation were purchased from POCH (Avantor, Poland).

### Other chemicals

In this study, we also used EDTA, Tris, mineral oil, graphite powder, and agarose, which were purchased from Sigma-Aldrich, Poland. For electrophoresis were used 6x loading buffer and 100bp DNA ladder purchased from Novagen, and SYBR GOLD dye possessed from DNA Gdańsk, Poland.

### SWV assay

All SWV measurements were run using Autolab PGSTAT12 potentiostat (Eco Chemie, The Netherlands), supported by GPES 4.9 software (EcoChemie, Netherlands). During all measurements, the following current parameters were kept: step potential of 10 mV, amplitude of 40 mV, and frequency of 50 Hz. The electrode set comprised a three-electrode: a working electrode, to which the tested nucleic acid fragments were attached by electrostatic attraction, a measurement electrode used to register the intensity of the electric current depending on the applied potential, and a reference electrode to normalize the current value registered between the working and measuring electrode. For this study used were a carbon paste electrode (CPE) as the working one, Ag/AgCl (3 M KCl) as the reference, and Pt wire as the measuring electrode. Reference and measuring electrodes were purchased from Mineral (Warsaw, Poland), and CPE was prepared according to the Oczkowski and Filipiak protocol [37] with further modifications [38–40]. The carbon paste was prepared by mixing a graphite powder with mineral oil in a ratio 7:3 (w/w). The resulting paste was then homogenized in small tube and packed into Teflon tube of 0.1 cm internal diameter. Electrical connection was supplied with a copper wire. After each measurement, the working electrode was regenerated by rubbing the outer layer of the working electrode (where the detection occurs) on a weighting paper to dispose oxidated analytes from the surface of CPE. Then new portion of carbon paste on CPE surface was manually applied making octal and circular movement on the frosted microscope slide. The smooth layer of the electrode warranted the homogenous surface for DNA detection and enhanced the repeatability of registered signals. The whole electrode set was stabilized with a monopod with height regulation. Measurements were performed in 1-ml wells of 24-well plate in the first well, the conditioning stage was performed, and then the electrode set was shifted to a second well with the tested DNA solution. The electrode washing process (only for protocol B) occurred in well 3, which was filled with the buffer used. Each well was provided with a magnetic stirring bar placed at the bottom. The stirring bar working at speed 200 rpm was employed to facilitate the migration of the tested DNA samples towards the detection layer of CPE. All measurements were carried out at ambient temperature.

In the study, we applied two different protocols based on our previous research [38, 41, 42]. The first procedure (protocol A) involved 60-second conditioning of the electrode set in pure buffer at the potential of +1.7 V. After that point, the background signal was registered. The electrode set was then transferred to the vessel with the sample, and the assayed DNA samples were deposited on the CPE surface by 120 s at the potential of +0.5 V. Next, SWV measurement was performed. Another procedure (protocol B) differed by transferring the electrodes after the deposition stage to another vessel where the unbonded DNA strands were removed by washing in buffer for 30 seconds under no-current conditions. After that additional stage, the SWV measurement ran. Each sample was measured minimum 8-fold to limit the number of potential errors. Then, the data were initially preprocessed using GPES software. First, the registered electric signal was smoothed using the Salvitzky-Golay algorithm to eliminate noises from the background. To facilitate the interpretation and calculation of the data, the background signal registered in the first stage of the SWV measurement cycle was subtracted from the sample signal. The result of the preprocessing stage was normalized data with baseline transformed to linear value Y = 0.

The ratio of guanine to adenine signal current (G/A) was used to compare the influence of selected factors (pH, temperature, H_2_O_2_) on changes in SWV signals of nucleic acid samples.

### Electrophoresis

Electrophoretic separation was performed for the electrochemically analyzed DNA samples in order to relate the signals recorded on the voltammograms to the image of the agarose gel. This allowed for the assessment of the degree of noticeability of damage to nucleic acid samples using both techniques. For electrophoresis, TBE buffer was used (pH 8.0, 89 mM Tris, 89 mM boric acid, 2 mM EDTA). Separation was performed on agarose gel (1%) in TBE buffer. The tested DNA samples were mixed with loading buffer 6x (Novazym, Poland) and inserted into the gel in the amount of 2.5 μL. The experiment was carried out using a compact M electrophoresis set with a Power Pack P25 power generator (Biometra, Germany). Electrophoresis ran for 1 hour at a potential of 80 V (4.5 V/cm). The gel was then stained with SYBR GOLD (1μl in10 mL TRIS-HCl, pH 7.4) and observed in UV light with wavelength range of 302–312 nm.

### Statistical analysis

The outstanding voltammograms were rejected, and for the rest, the peak analysis was performed to identify their locations and current. The signal currents located at the same potential value were then compared in terms of their mean current (A) and relative standard deviation (RSD). RSD was calculated according to the formula:

$$RSD=\;\frac{\sigma }{N}$$

Where: σ–standard deviation, N—number of measurements.

In addition, the analysis of variance (ANOVA) and Tukey post-hoc test were performed to illustrate significant differences between the analyzed DNA samples, pH values and tested procedures. All this information is presented in the supplementary file attached to this article.

All formal analyzes were made with the help of Origin Pro software (Origin, Germany).

## Results and discussion

**Fig 1:**
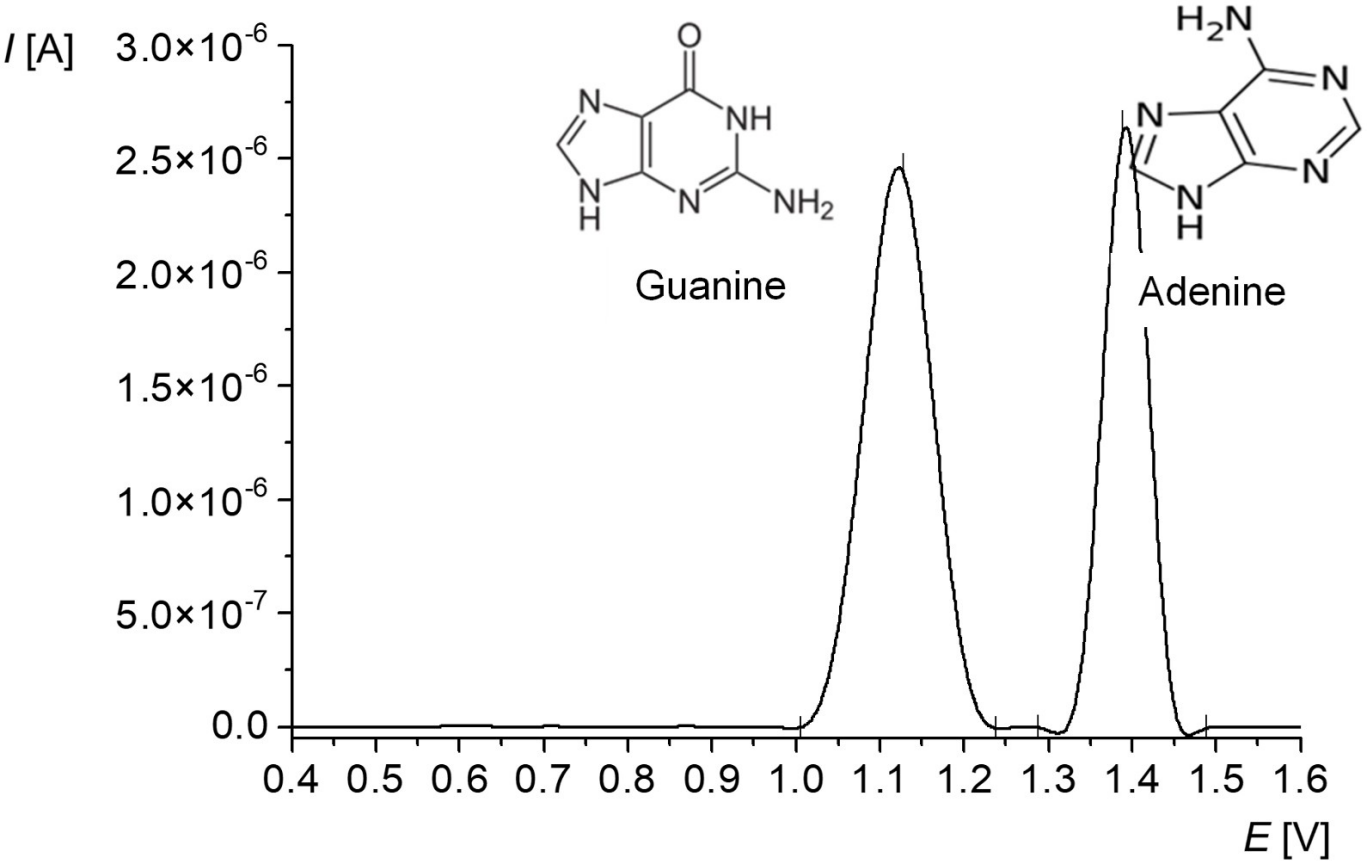
SWV voltammogram of 20 μg/mL ctDNA in acetate buffer.

Voltammetric analysis of nucleic acid delivers characteristic two signals (Fig 1). The first peak located in the potential range from +0.9 to +1.1 V is a signal of guanine, while the other, in the *E* range from +1.2 to +1.4 V, is adenine [41]. No signals for cytosine and thymine have been registered so far. However, both are complementary to guanine and adenine, respectively; thus, their electrochemical determination does not seem to be essential to assess the damage in the evaluated material.

Guanine and adenine peaks high, their area and position on the potential axis are characteristic of the analyzed nucleic acid sample and the measurement parameters used, in particular the type of working electrode and the buffer used, especially its pH value [22, 43]. For native dsDNA, hydrogen bonds and the double helical structure are a sterical hindrance, which limits the contact of the nucleic bases with the detection layer of CPE [38, 43]. Thus, the risk of oxidation is lower for dsDNA. Since the analyte is irreversibly oxidized during the SWV measurement, no bias agents are generated in the sample solution, and the solution can be retested in the next repetitions. Additionally, the SWV technique enabled detection of DNA damage at the lowest concentration of 10 nM, allowing to observe changes not detected by using of other methods [41, 44].

### Voltammetric analysis of oligonucleotide modified with 8-oxoguanine

**Fig 2:**
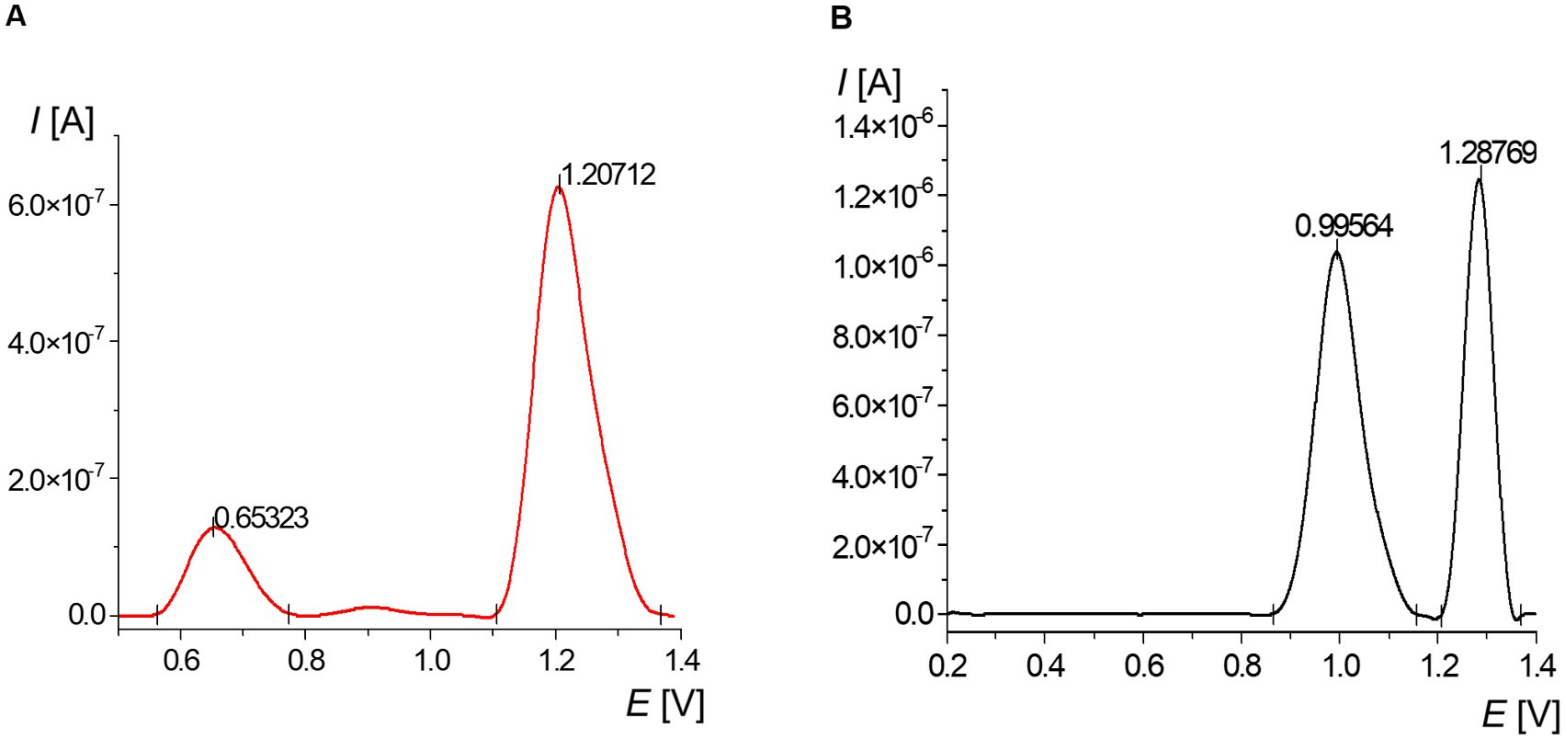
Average voltammogram of the 10 μM 5’GTCAACTTCCGTACCGAGC fragment in phosphate buffer: Modified by 8-oxoguanine (A) and without modification (B).

To facilitate the analysis of oxidative changes in SWV signals of DNA subjected to thermal denaturation and oxidative stress, measurements were initially performed using two oligonucleotides with the 5’GTCAACTTCCGTACCGAGC sequence, one of which was modified with one 8-oxoguanine (Fig 2).

For the non-modified oligonucleotide, two peaks were registered: guanine with a potential +1.00 V and adenine with a maximum at +1.28 V (Fig 2b). The parameters of these signals were included in Table 1. The calculated ratio of guanine and adenine peak current was 0.83.

**Table 1:** Detailed parameters of electrochemical signals detected for non-modified oligonucleotide.

Parameter	Nitrogen base	*E* (V)	*I* (nA)
**Mean**	Guanine	1.00	1,045.07
**RSD [%]**		**0.00**	**19.54**
**Mean**	Adenine	1.28	1,259.65
**RSD [%]**		**0.45**	**20.46**
**N**	**Both**	**3**	
**G/A ratio**		**0.83**	

The measurement performed for an oligonucleotide in which only one guanine was replaced by 8-oxoguanine showed a surprising result, due to the lack of a guanine signal (Fig 2a). The presence of 8-oxoG was recorded in the potential range from +0.6 to +0.8 V, with a maximum at +0.67 V (Table 2). The signal of adenine shifted to lower potential compared to the non-modified oligonucleotide, and was located at +1.21 V. It was not possible to determine the ratio of G/A signals, but the ratio of 8-oxoG/A was 0.28.

**Table 2:** Detailed parameters of electrochemical signals detected for the 8-oxoG modified oligonucleotide.

Parameter	Nitrogen base	*E* (V)	*I* (nA)
**Mean**	8-oxoguanine	0.67	169.93
**RSD [%]**		**0.76**	**21.7**
**Mean**	Adenine	1.21	609.99
**RSD [%]**		**0.38**	**10.2**
**N**	**Both**	**10**	
8-oxoG/A ratio		**0.28**	

Adenine in the unmodified oligonucleotide was more labile to oxidation than in the fragment with 8-oxoG. This situation may be explained by a further favorable oxidation of oxidized analogues of nucleobases compared to non-oxidized ones [45]. Also observed was a positive relationship for adenine between registered the potential value and the peak current. This relation could be applied to monitor DNA damage exposed to oxidation stress. However, this hypothesis could be validated on the basis of diverse and numerous nucleic materials.

The analysis of variance (S1 File) showed that modification of guanine with 8-oxoG led to significant changes in the registered signals.

### Effect of thermal treatment on DNA damages

To observe the damages induced by thermal treatment, the dsDNA aliquots of calf thymus in phosphate buffer (pH 7.0) and acetate buffer (pH 4.7) were melted at 100°C for 1 h. For this part of the study, we applied two separate protocols: without washing the electrode set after dsDNA deposition (A) and with it (B).

#### DNA samples dissolved in phosphate buffer (pH 7.0)

**Fig 3:**
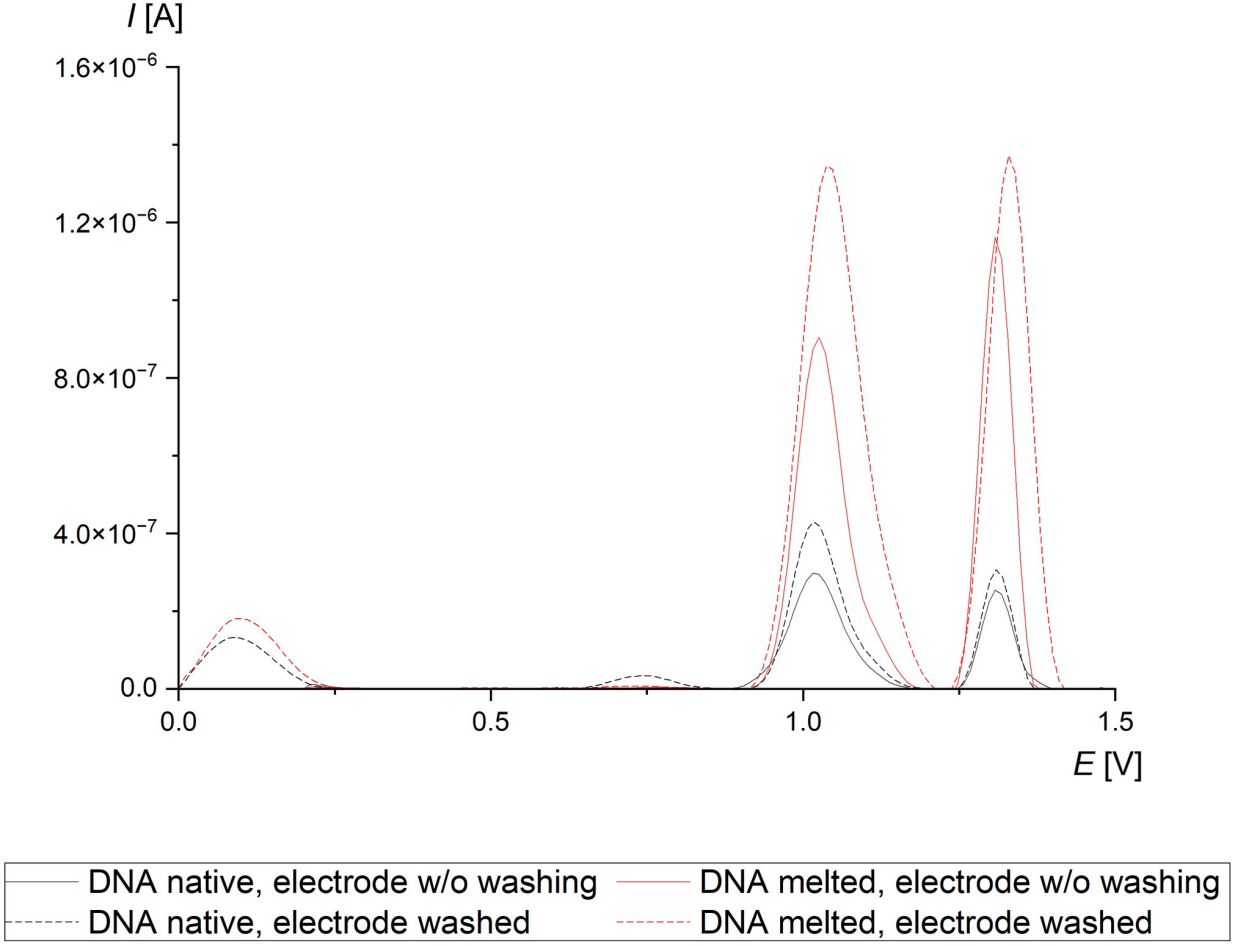
Average voltammograms of ctDNA in phosphate buffer (pH 7.0).

As a reference, electrochemical signals were registered for dsDNA not subjected to the thermal treatment (Fig 3). Both signals of purine bases were visible on the voltammogram. The guanine signal was located between +0.9 and +1.2 V potentials, while the adenine signal was between +1.2 and +1.4 V (Table 3). These parameters cover the positions registered in our previous studies [38, 42].

**Table 3:** Detailed parameters of electrochemical signals detected for 10 μg/mL calf thymus DNA in phosphate buffer (pH 7.0).

Protocol	DNA state	Parameter	Nitrogen base	*E* (V)	*I* (nA)
**A**	**Native**	Mean	Guanine	1.02	309.84
		RSD [%]		**1.13**	**43.19**
		Mean	Adenine	1.32	281.26
		RSD [%]		**1.17**	**57.11**
		N	**Both**	**13**	
		**G/A ratio**		**1.10**	
	**Melted**	Mean	Guanine	1.02	904.71
		RSD [%]		**0**	**59.86**
		Mean	Adenine	1.31	1,165.53
		RSD [%]		**0.40**	**62.17**
		N	**both**	**12**	
		**G/A ratio**		**0.78**	
**B**	**Native**	Mean	Guanine	1.02	462.46
		RSD [%]		**0.63**	**49.55**
		Mean	Adenine	1.31	352.27
		RSD [%]		**0.68**	**58.66**
		N	**Both**	**8**	
		**G/A ratio**		**1.31**	
	**Melted**	Mean	Guanine	1.04	1,351.19
		RSD [%]		**0.52**	**47.80**
		Mean	Adenine	1.33	1,401.89
		RSD [%]		**0.81**	**41.23**
		N	**Both**	**15**	
		**G/A ratio**		**0.96**	

The denatured DNA had two electrochemical signals, located respectively between +0.9 and +1.2 V, and between +1.2 and +1.4 V (Fig 3). In contrast to the native dsDNA, it was observed that the melted DNA was characterized by a higher intensity of adenine than guanine signal (Table 3). This inconsistency may originate from partial guanine oxidation during the melting process [20]. The low signal located between +0.6 and +0.8 V supports this hypothesis and confirms 8-oxoguanine formation. Then 8-oxoguanine can be further oxidized to form non—electroactive products [46].

The non-deviated potential value of guanine clearly shows that no changes affected the registered voltammogram. High signal current may result from random environmental factors or heterogeneous DNA adsorption on the CPE layer. Nonetheless, these factors play no significant role in the context of the qualitative analysis presented in this study. Adenine differed in its potential range and peak intensity more noticeably, analogously to that in the case of unmelted ctDNA. However, the deviation of the adenine potential in melted DNA is lower, which could be an effect of more feasible binding of ssDNA to CPE.

As a result of the facilitated adsorption of ssDNA on CPE, both registered peaks had higher intensity. The adenine signal is also thinner due to base pairs adjacent to adenosine are partially oxidized, and thus are less susceptible to further oxidation occurring in the measurement process. Adenine has the lowest reduction potential among all nucleobases and may oxidize temporarily during melting. It is consistent with the theorem that oxidized bases may be reduced by oxidizing the other ones [45, 47]. In the mentioned model, the condition for such reaction is the lower reducing potential (*E*_red_) of the nucleophile. As 8-oxoguanine has a lower *E*_red_ than adenine, the oxidation shifted toward the first.

During the melting process, guanine is more labile to attack by reactive oxygen species (ROS). Since guanine is the strongest electron donor, its oxidation continues until the compound is received without conjugated double bonds or biological activity [47–49]. Therefore, the guanine signal dropped after melting and no forms of oxidized by-products were present. However, the conclusions from this part of the study should be confirmed by performing quantitative HPLC-MS analysis of the nucleotides and their derivatives.

The addition of CPE washing to the protocol helped visualize the oxidized derivatives of guanine, which signal covers 8-oxoG registered in the prior stage of the study (potential from +0.6 to +0.8 V) (Fig 3).

The current intensity values of both peaks are similar to those registered using the protocol A, but washing the electrodes warranted higher DNA signals (Table 3). The signal located at *E* +0.75 V is also stronger. Moreover, the additional washing of the electrode helped visualize significant differences between DNA in native and melted states, despite the high internal variance for both samples (S1 and S2 Files). The higher G/A values noted for both samples registered in protocol B, show that DNA sample with oxidized guanine is adsorbed weaker than unchanged DNA, and is more susceptible to desorption from the CPE layer.

This may be the reason of no oxidized forms noticed for the melted DNA, with the help of protocol B (Fig 3). The ratio between guanine and adenine signal changed, but this may be random and not affected by any oxidative damage. The lower decrease in the guanine signal for the protocol B could be affected by the differences between the two applied protocols (Table 3). In the protocol A, oxidized are all nucleotides in the vicinity of the working electrode, not only the adsorbed ones. Under such conditions, the conductivity measured could be a sum of currents participating in redox reactions between CPE and nucleotides and electrons, and charges creating hydration coats and hydrogen bonds between the DNA-CPE complex and DNA dispersed in the buffer. Another possible factor could be H_2_O_2_ or ROS generated during the gradual growth of the potential set and the bias in the shape of the voltammogram.

#### DNA samples dissolved in acetate buffer (pH 4.7)

For illustration of the changes, we decided to perform an analysis according to protocol B, because of its better registration of oxidized derivatives of guanine. The application of the protocol A at this stage of the study resulted in various results, which may hardly pose any scientific value.

**Fig 4:**
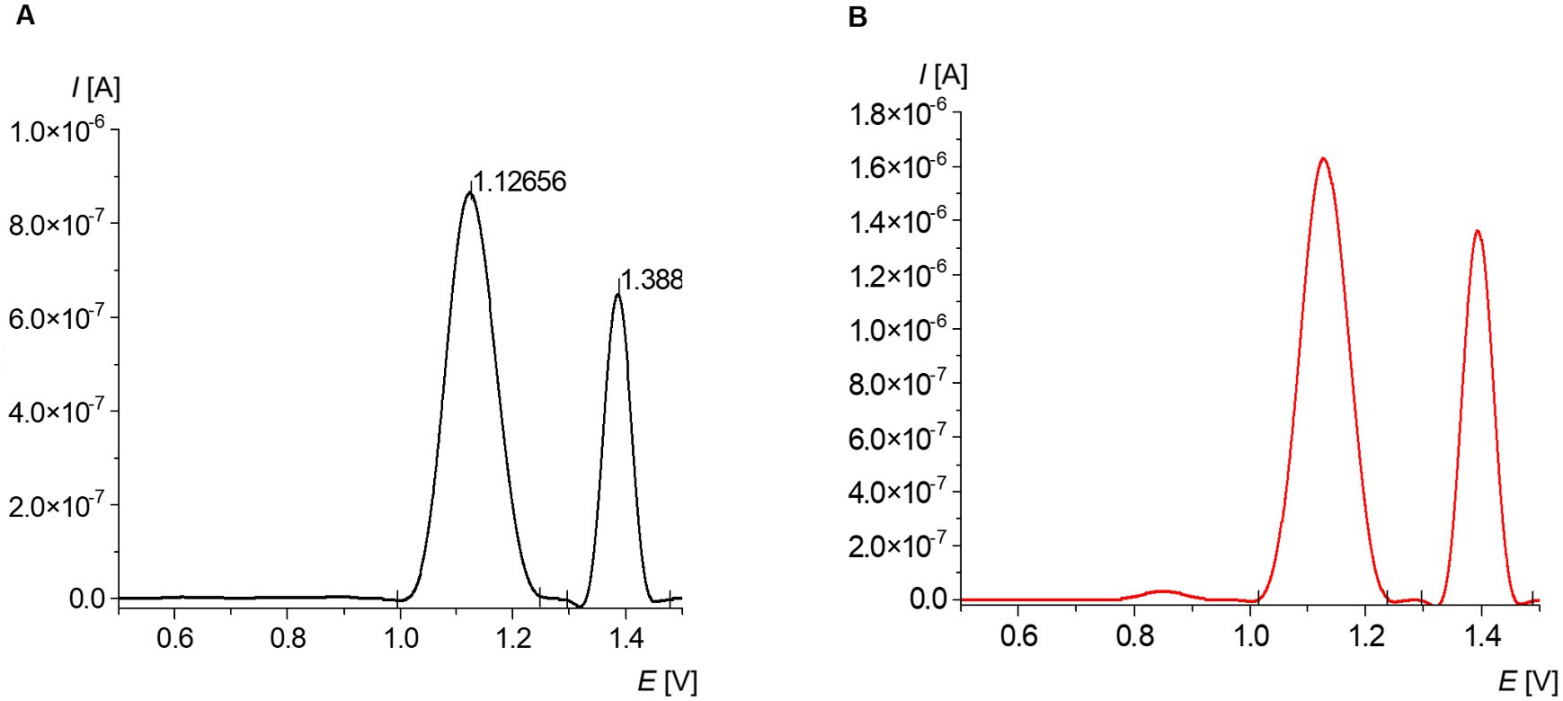
Average voltammogram of 10 μg/mL calf thymus dsDNA in acetate buffer (pH 4.7), registered using washed CPE after the deposition. A—native DNA; B—melted DNA.

The collected results show that the studied dsDNA is dominated by GC base pairs over AT (Fig 4A). Analogical results were noted for the sample dissolved in phosphate buffer. The guanine signal ranged from +1.0 to +1.03 V, while the adenine signal was present between +1.3 and +1.5 V (Table 4).

**Table 4:** Detailed parameters of electrochemical signals detected for 10 μg/mL calf thymus dsDNA in acetate buffer (pH 4.7), registered according to protocol B.

DNA	Parameter	Nitrogen base	*E* (V)	*I* (nA)
**Native**	Mean	Guanine	1.12	875.56
	RSD [%]		**0.52**	**27.07**
	Mean	Adenine	1.39	663.13
	RSD [%]		**0.00**	**6.13**
	N	**Both**	**4**	
	**G/A ratio**		**1.32**	
**Melted**	Mean	Guanine	1.13	1,649.11
	RSD [%]		**0.36**	**48.01**
	Mean	Adenine	1.39	1,412.21
	RSD [%]		**0.40**	**52.90**
	N	**Both**	**6**	
	**G/A ratio**		**1.17**	

The adenine signal deviated less than that for guanine, contrasting with the data received for the sample dissolved in phosphate buffer. The protocol B and buffer used in this study also positively affected the current of the resulting peaks. The results show that the measurement performed for the sample dissolved in acetate buffer provided higher selectivity in the detection of dsDNA compared to the measurement of samples dissolved in phosphate buffer. This indicates that sample diluting in acidic conditions may facilitate quantitative determination of genetic material.

After thermal denaturation was performed, no changes in the location of the DNA signal were observed (Fig 4B). The voltammogram illustrates that the guanine/adenine ratio changed by 11% after melting, which is not a noticeable change. Changes in the guanine signal correspond to a low peak registered between the +0.8 and +0.9 V (Table 4). Total damage registered in the form of this peak may show that the oxidative changes in DNA melted under acidic conditions are less dynamic and complex.

Compared to native DNA, the melted one differed more in the peak potential, which may be induced by thermal damage (Figs 3 and 4). Interestingly, the guanine signal from ssDNA is more stable than adenine, which was also observed in another study [41]. However, the thermal effect is not closely related to the intensity of the reversive dsDNA peaks noted for the acetate buffer. These discrepancies may be explained by the fact that the repeatability of the method depends on the number of adsorbed oxidizable compounds on the CPE layer.

In dsDNA, electron transfer is limited by the steric hindrance [47, 50]. Meanwhile, electron transfer can occur between adjacent nucleobases. Thus, adenine can change its role from an electrophile to a nucleophile and emit an additional electron stream that could affect the signal registered in the SWV method [51].

Signals registered for the sample dissolved in phosphate buffer are lower and shifted toward a lower potential. On the basis of the obtained results, it can be concluded that in acidic pH conditions, the electrochemical detection of DNA oxidation products is more sensitive, which may be useful, for example, when detecting cancer markers. What is more, during the melting process in acidic condition, the majority of guanine oxidized, which was observed as a decline in G/A ratios for both studied protocols by 35 and 27%, respectively. The oxidized derivatives of guanine can affect the data collected using other methods and bias their results [52, 53].

### Oxidation-induced damage to DNA

As an oxidation factor, we applied H_2_O_2_ with a final concentration of 1.5% in the prepared sample. The nucleotides were exposed to the oxidative effect of H_2_O_2_ by 300 s. The tested material was calf thymus DNA dissolved in phosphate buffer, analogously to that in the previous part of the research. At this stage, we ran SWV analyses using the B protocol only.

In order to observe changes in the DNA structure resulting from the action of H_2_O_2_, measurements were made for native dsDNA at ambient temperature and for ssDNA after the melting process.

**Fig 5:**
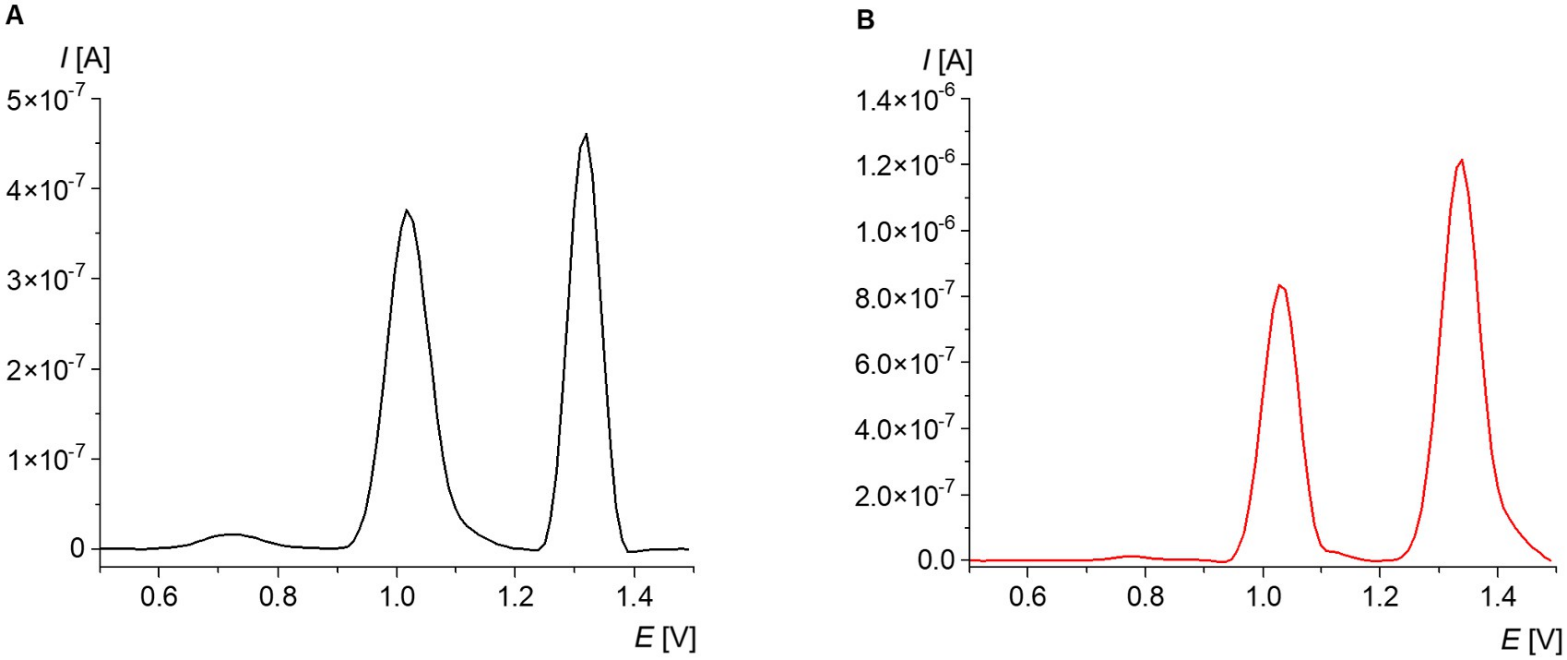
Average voltammogram of 10 μg/mL calf thymus ds DNA exposed to H_2_O_2_ for 1 h. A—native DNA; B—melted DNA.

For DNA exposed to H_2_O_2,_ characteristic signals of oxidized derivatives of guanine can be observed, that are located between +0.6 and +0.8 V (Fig 5). In Fig 5A, the characteristic bulge of the guanine signal is also seen, which could be an overlapped signal of adenine oxidized derivatives [54]. The drop in G/A ratio suggests that the dose of H_2_O_2_ added to the DNA sample oxidized nearly 40% of the guanine detected in the previously tested ctDNA sample (Tables 3 and 5).

**Table 5:** Detailed parameters of electrochemical signals detected for 10 μg/mL calf thymus dsDNA in phosphate buffer (pH 7.0), exposed to H_2_O_2_ for 1 h.

Parameter	Nitrogen base	*E* (V)	*I* (nA)
**Mean**	Guanine	1.02	378.43
**RSD [%]**		**0.40**	**51.88**
**Mean**	Adenine	1.31	480.73
**RSD [%]**		**0.80**	**47.20**
**N**	**Both**	**6**	
**G/A ratio**		**0.79**	

Consistent with the Van’t-Hof rule, the oxidation rate should be exponentially higher during the melting process than it was observed at room temperature. The G/A ratio declined by 30%, compared to the melted ctDNA without the addition of H_2_O_2_, and almost halved compared to the dsDNA (Tables 3 and 6). However, the current of the peak characteristic for 8-oxoG and other oxidized derivatives declined after the melting process. Therefore, the oxidation decay of guanine was complete and resulted in the generation of non-active products (Table 5) [46]. In addition, the bulge part of the guanine peak decreased, which may be an effect of adenine decay to non-active products. However, the analysis of variance showed that the differences between the oxidized DNA in double- and single-stranded form were insignificant (S1 File).

**Table 6:** Detailed parameters of electrochemical signals detected for 10 μg/mL melted ctDNA in phosphate buffer (pH 7.0), exposed to H_2_O_2_ for 1 h.

Parameter	Nitrogen base	*E* (V)	*I* (nA)
**Mean**	Guanine	1.027	833.97
**RSD [%]**		**0.00**	**37.53**
**Mean**	Adenine	1.34	1,238.11
**RSD [%]**		**0.43**	**14.71**
**N**	**Both**	**3**	
**G/A ratio**		**0.67**	

The new irregularity in Fig 5B is a bulge of adenine at the higher potential window that is related to feasible oxidation of thymidine and cytosine. Meanwhile, the guanine and adenine peaks grew, probably due to the stronger DNA adsorption onto the CPE surface (Table 6) [55].

### Electrophoresis results

**Fig 6:**
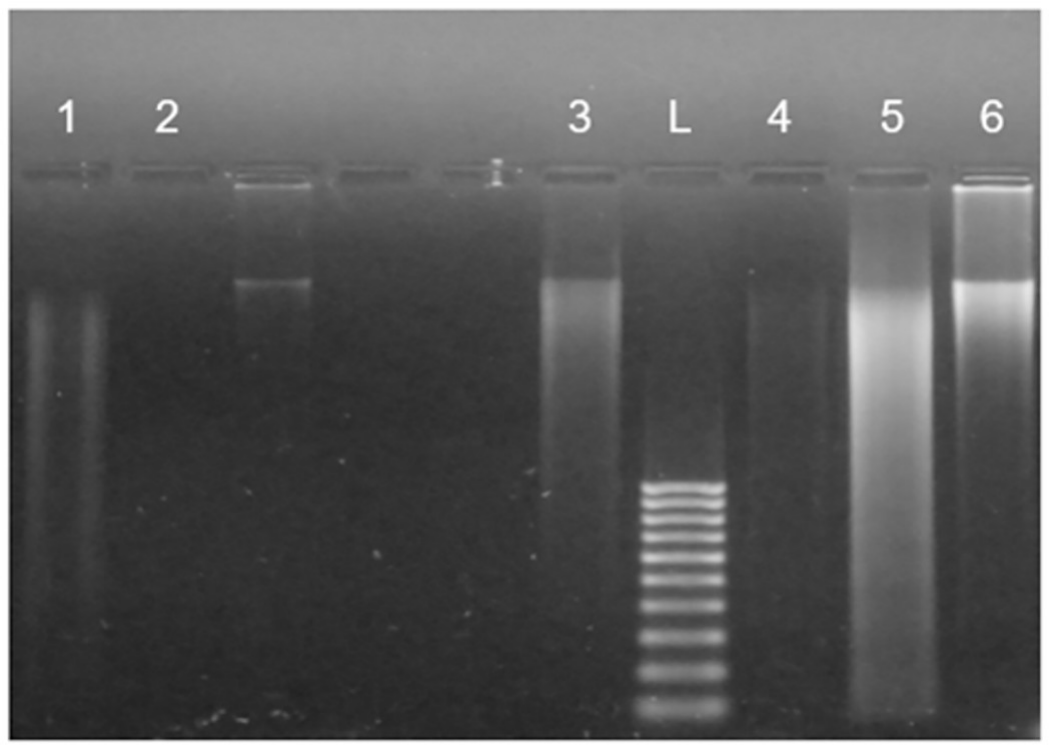
Electrophoresis of tested DNA samples. **1**: double-stranded ctDNA exposed to H_2_O_2_; **2**: melted ctDNA exposed to H_2_O_2_; **3**: melted ctDNA at pH 7.0; **4**: double-stranded ctDNA at pH 7.0; **5**: melted ctDNA at pH 4.7; **6**: double-stranded ctDNA at pH 4.7;** L**–DNA ladder.

As SWV is still considered a novel and uncommon technique, we ran electrophoretic separation of tested DNA samples in this study as a reference method (Fig 6). Novak et al. [56] indicated that temperature-induced damage to DNA can be observed in electrophoresis gels. However, the authors of the referred study reached such a conclusion only on the basis of samples diluted in neutral pH buffer.

DNA electrophoretic separation presented in Fig 6 confirmed the results of the SWV study. Exposure to the added source of oxidative stress to native dsDNA resulted in hydrolysis of the strands. DNA strand breaks leading to its fragmentation were observed in the electropherograms in the form of blurred bands. In the sample subjected to oxidative stress, no traces of DNA were observed on the agarose gel. This may lead to the conclusion that the decay of ssDNA proceeded so intensely that the obtained fragments were so short that they cannot be noted in the set electrophoresis conditions.

Melted DNA, not treated with H_2_O_2_, was more visible on the gel compared to the sample subjected to oxidative stress. However, it can be seen that DNA denaturation causes strand breaks because the bands become more blurry after denaturation.

Under acidic conditions, a higher blur of the dsDNA fragment was observed than at neutral pH, which may result from a higher hydrolysis of the strand promoted under acidic conditions. It can be seen that the hydrolysis rate of dsDNA was lower than that of melted DNA. In the case of dsDNA, longer fragments were observed on the gel. Although DNA fragmentation can be observed on the gel after electrophoresis, it cannot be used to assess nucleobase damage.

## Conclusions

The use of electrochemical analysis of nucleic acid samples allowed to identify guanine derivatives formed as a result of oxidative stress. Our preliminary study showed that SWV can identify the presence of oxidized guanine derivatives in the oligonucleotide fragment, and the +0.8 V signal corresponds to the presence of 8-oxoG. This finding allowed on further analysis of DNA damage occurring after denaturation and exposure to oxidative stress.

We also validated if additional washing of CPE electrode after DNA electrostatic deposition could increase its response rate. For native dsDNA, the ratio of guanine and adenine signal (G/A) was 1.10 without electrode washing (protocol A) and 1.31 after washing (protocol B). After the melting process, the G/A ratio decreased to 0.78 (protocol A) and to 0.96 (protocol B), what demonstrated guanine partial oxidation. DNA damage was registered stronger using the protocol A compared to B, which confirm that oxidized DNA binds weaker to the CPE surface and is desorbed during the electrode washing process. The results measured using the first protocol showed 30% oxidized guanine in the tested sample, while the other protocol noted 27% of its oxidation. Since protocol A might bias the final results by additional registration of side reactions of nucleotides not attached to the CPE layer, the more reliable results could be collected using protocol B.

For the acidic conditions received was a similar value of G/A ratio– 1.32. This leads to the conclusion that pH had no effect on the intensity of the nucleobase signal. After thermal treatment, no meaningful changes in the G/A signal ratio were observed. It decreased by 11% to 1.17 level, but the presence of oxidized derivatives was recorded in the potential range from +0.8 to +1.0 V. Since the oxidized derivatives were not visible for dsDNA, it can be assumed that the oxidation of guanine and adenine occurred in the acetate buffer with lower kinetics than in the phosphate one. The addition of the oxidizing agent, H_2_O_2_, showed with higher strength the oxidation changes in melted DNA. Furthermore, dsDNA exposed to H_2_O_2_ had higher G/A ratio (0.79) than for DNA denatured in the melting process (0.67).

The obtained results indicate that, depending on the type of performed nucleic acid analyses, the solvent and its pH should be selected appropriately, e.g., volumetric and quantitative experiments, such as density gradient, may require dissolving the DNA in an acidic pH, e.g. in an acetate buffer, to prevent strand cleavage. On the other hand, sequencing methods, polymerase chain reaction (PCR) and blotting would require DNA dissolved in buffer with a neutral pH such as phosphate, to decrease the probability of nucleobases oxidation.

## Supporting information

S1 Fig Raw image of the electrophoresis presented in Fig 6. **1**: double stranded ctDNA exposed to H_2_O_2_; **2**: melted ctDNA exposed to H_2_O_2_; 3: melted ctDNA at pH 7.0; **4**: double stranded ctDNA at pH 7.0; **5**: melted ctDNA at pH 4.7; **6**: double stranded ctDNA at pH 4.7; **L**—100 bp DNA ladder (Novagen, Poland); **N**—sample not include d in this study and not mentioned in Fig 6; X lane not included in Fig 6. The DNA fragments were visualized by UV fluorescence after being stained with SYBR GOLD dye.
(PDF)

S1 File Analysis of variance (ANOVA) between the tested oligonucleotides, protocols and buffers applied. The level of confidence (α) was 0.05.
(DOCX)

S2 File Raw data of the SWV study. (XLSX)
