# Supplementary material for: May DNA analyses be biased by hidden oxidative damage? Voltammetric study of temperature and oxidation stress effect
Source: PLoS One. 2024 Jun 14;19(6):e0305590. doi: 10.1371/journal.pone.0305590 (PMC11178200; doi:10.1371/journal.pone.0305590)
Supplement: S1 Fig — 1: double stranded ctDNA exposed to H2O2; 2: melted ctDNA exposed to H2O2; 3: melted ctDNA at pH 7.0; 4: double stranded ctDNA at pH 7.0; 5: melted ctDNA at pH 4.7; 6: double stranded ctDNA at pH 4.7; L—100 bp DNA ladder (Novagen, Poland); N—sample not include d in this study and not mentioned in Fig 6; X lane not included in Fig 6. The DNA fragments were visualized by UV fluorescence after being stained with SYBR GOLD dye. (PDF) [file pone.0305590.s001.pdf]

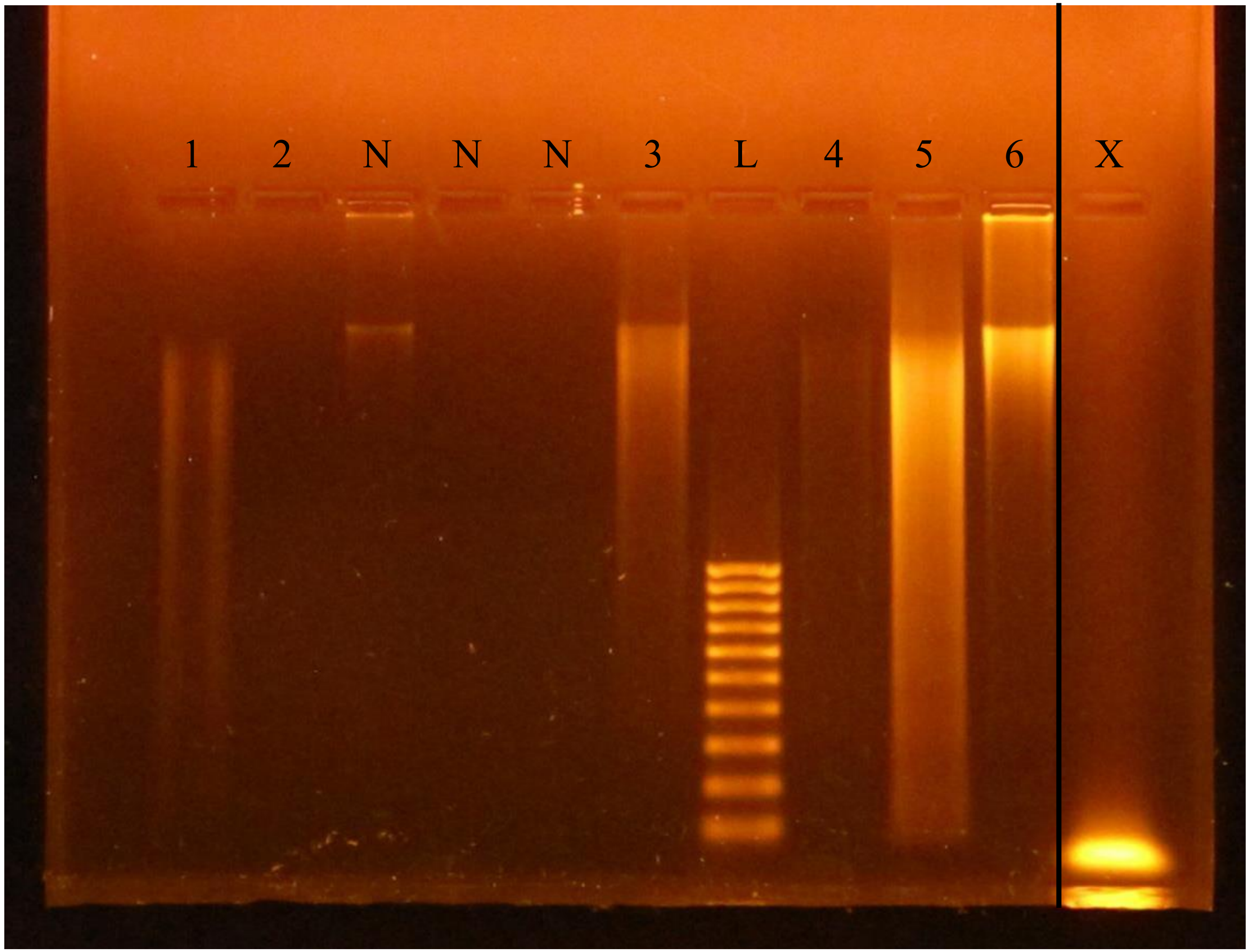

Figure S1. Raw image of the electrophoresis presented in Figure 6.. **1**: double-stranded ctDNA exposed to H_2O_2; **2**: melted ctDNA exposed to H_2O_2; **3**: melted ctDNA at pH 7.0; **4**: double-stranded ctDNA at pH 7.0; **5**: melted ctDNA at pH 4.7; **6**: double-stranded ctDNA at pH 4.7; **L** – 100 bp DNA ladder (Novagen, Poland); **N** – sample not included in this study and not mentioned in Figure 6; **X** – lane not included in Figure 6. The DNA fragments were visualized by UV fluorescence after being stained with SYBR GOLD dye.
