# Supplementary material for: May DNA analyses be biased by hidden oxidative damage? Voltammetric study of temperature and oxidation stress effect
Source: PLoS One. 2024 Jun 14;19(6):e0305590. doi: 10.1371/journal.pone.0305590 (PMC11178200; doi:10.1371/journal.pone.0305590)
Supplement: S1 File — The level of confidence (α) was 0.05. (DOCX) [file pone.0305590.s002.docx]

**Supplementary material**

This supplementary materials supports the date presented in the main part of the article with analysis of variance (ANOVA) between the tested oligonucleotides, protocols and buffers applied. The level of confidence (α) was 0.05

**Comparison of BAR-3 and BAR-3 modified with 8-oxoguanine**

***Overall ANOVA***

|  | DF | Sum of Squares | Mean Square | F Value | Prob>F |
| --- | --- | --- | --- | --- | --- |
| Model | 1 | 5.5662E-13 | 5.5662E-13 | 8.63555 | 0.00362 |
| Error | 238 | 1.53407E-11 | 6.44568E-14 |  |  |
| Total | 239 | 1.58973E-11 |  |  |  |
| **Null Hypothesis:** The means of all levels are equal.  **Alternative Hypothesis:** The means of one or more levels are different**.** At the 0.05 level, the population **means are significantly different.** | | | | | |

**Differences between analytical procedures and form of DNA tested**

***Overall ANOVA* *between native and melted ctDNA using two different protocols***

|  | DF | Sum of Squares | Mean Square | F Value | P Value |
| --- | --- | --- | --- | --- | --- |
| A) CPE unwashed after deposition | 1 | 2.24199E-13 | 2.24199E-13 | 3.75155 | 0.05329 |
| B) CPE washed after deposition | 1 | 1.85032E-12 | 1.85032E-12 | 30.96166 | <0.0001 |
| Interaction | 1 | 9.2403E-14 | 9.2403E-14 | 1.54619 | 0.21424 |
| Model | 3 | 2.27997E-12 | 7.5999E-13 | 12.71701 | <0.0001 |
| Error | 534 | 3.19127E-11 | 5.97617E-14 |  |  |
| Corrected Total | 537 | 3.41927E-11 |  |  |  |
| At the 0.05 level, the population **means of Factor A are not significantly different.** At the 0.05 level, the population **means of Factor B are significantly different.** At the 0.05 level, **the interaction between Factor A and Factor B is not significant .** | | | | | |

***Tukey post-hoc test***

| CPE unwashed after deposition | CPE washed after deposition | Mean | Groups | | |
| --- | --- | --- | --- | --- | --- |
| Melted DNA | Melted DNA | 1.98798E-7 | A |  |  |
| Native DNA | Melted DNA | 1.31366E-7 | A | B |  |
| Melted DNA | Native DNA | 5.44554E-8 |  | B | C |
| Native DNA | Native DNA | 3.97529E-8 |  |  | C |
| Means that do not share a letter are significantly different. | | | | | |

**Differences between pH values and form of DNA tested**

***Overall ANOVA***

|  | DF | Sum of Squares | Mean Square | F Value | P Value |
| --- | --- | --- | --- | --- | --- |
| pH 7.0 | 1 | 1.61453E-12 | 1.61453E-12 | 8.54115 | 0.00361 |
| pH 4.7 | 1 | 4.4459E-14 | 4.4459E-14 | 0.2352 | 0.62788 |
| Interaction | 1 | 2.32081E-12 | 2.32081E-12 | 12.2775 | 4.94185E-4 |
| Model | 3 | 4.00212E-12 | 1.33404E-12 | 7.05731 | 1.1535E-4 |
| Error | 574 | 1.08503E-10 | 1.89029E-13 |  |  |
| Corrected Total | 577 | 1.12505E-10 |  |  |  |
| At the 0.05 level, the population **means of Factor A are significantly different.** At the 0.05 level, the population **means of Factor B are not significantly different.** At the 0.05 level, **the interaction between Factor A and Factor B is significant .** | | | | | |

***Tukey post-hoc test***

| pH 7.0 | pH 4.7 | Mean | Groups | |
| --- | --- | --- | --- | --- |
| Melted DNA | Native DNA | 2.87003E-7 | A |  |
| Native DNA | Melted DNA | 1.98798E-7 | A |  |
| Melted DNA | Melted DNA | 1.77759E-7 | A | B |
| Native DNA | Native DNA | 5.44554E-8 |  | B |
| Means that do not share a letter are significantly different. | | | | |

**Effect of melting on stability of oxidized ctDNA**

***Overall ANOVA***

|  | DF | Sum of Squares | Mean Square | F Value | Prob>F |
| --- | --- | --- | --- | --- | --- |
| Model | 1 | 7.0713E-16 | 7.0713E-16 | 0.0665 | 0.79669 |
| Error | 296 | 3.14768E-12 | 1.0634E-14 |  |  |
| Total | 297 | 3.14839E-12 |  |  |  |
| **Null Hypothesis:** The means of all levels are equal. **Alternative Hypothesis:** The means of one or more levels are different.  At the 0.05 level, the population **means are not significantly different.** | | | | | |

***Tukey pos-hoc test***

| Form | Mean | Groups | |
| --- | --- | --- | --- |
| Native | 5.67466E-8 | A | |
| Melted | 5.36658E-8 | A | |
| Means that do not share a letter are significantly different. | | |  |
